# A case of a patient receiving combination therapy with paclitaxel plus bevacizumab and adoptive activated αβ T‐cell immunotherapy in advanced breast cancer

**DOI:** 10.1111/tbj.14108

**Published:** 2020-12-05

**Authors:** Takaaki Masuda, Atsushi Nonami, Fumiaki Tanaka, Yuki Ando, Masatoshi Eto, Koshi Mimori

**Affiliations:** ^1^ Department of Surgery Kyushu University Beppu Hospital Beppu Japan; ^2^ Center for Advanced Medical Innovation Kyushu University Fukuoka Japan; ^3^ Tanaka Breast Surgery Internal Medicine Clinic Beppu Japan

**Keywords:** adoptive immunotherapy, bevacizumab, breast cancer, αβ T‐cell

Immunotherapy is now standard for many malignancies. In breast cancer (BC), the checkpoint inhibitor anti‐PD‐L1, which blocks immunosuppressive effects of tumors on T cells, is available only for patients with triple‐negative PD‐L1–positive advanced BC.^1^

Activated αβ T‐cell immunotherapy (ATI) is a nonspecific adoptive T‐cell therapy using activated αβ T cells.^2^ Activated αβ T‐cell immunotherapy has proven effective in various malignancies without serious side effects.^3^

We describe below a case of advanced hormone receptor (HR)–positive BC benefitting from combination therapy using ATI with paclitaxel (PTX) plus the anti‐vascular endothelial growth factor (VEGF) antibody bevacizumab (BV).

In 2003, a 27‐year‐old woman underwent partial mastectomy with axillary lymph node dissection for an HR‐positive HER2‐negative infiltrating ductal BC (pT1c, pN1a, M0), Then, she received adjuvant chemotherapy (epirubicin/cyclophosphamide [EC]), hormone therapy with luteinizing hormone‐releasing hormone (LHRH) analog and tamoxifen in addition to irradiation of the affected breast and infraclavicular nodes. From 2009, she was treated for multiple bone metastases with hormone therapy using an LHRH analog, bilateral oophorectomy, and aromatase inhibitor letrozole followed by high‐dose toremifene. In July 2018, she visited our hospital for recurrent BC with multiple lung and bone metastases with a bad cough.

From August 2018, she received PTX + BV with 3 courses, hormone therapy with fulvestrant plus CDK4/6 inhibitor Palbociclib, followed by EC. Starting in August 2019 (Day 0, Figure 1A), we began biweekly PTX + BV, which was still effective. On Day 89, CT revealed progressive disease (lung, liver, and bone metastases with pleural effusion and ascites). Vital signs were stable, though she experienced abdominal bloating and difficulty breathing.

**FIGURE 1:**
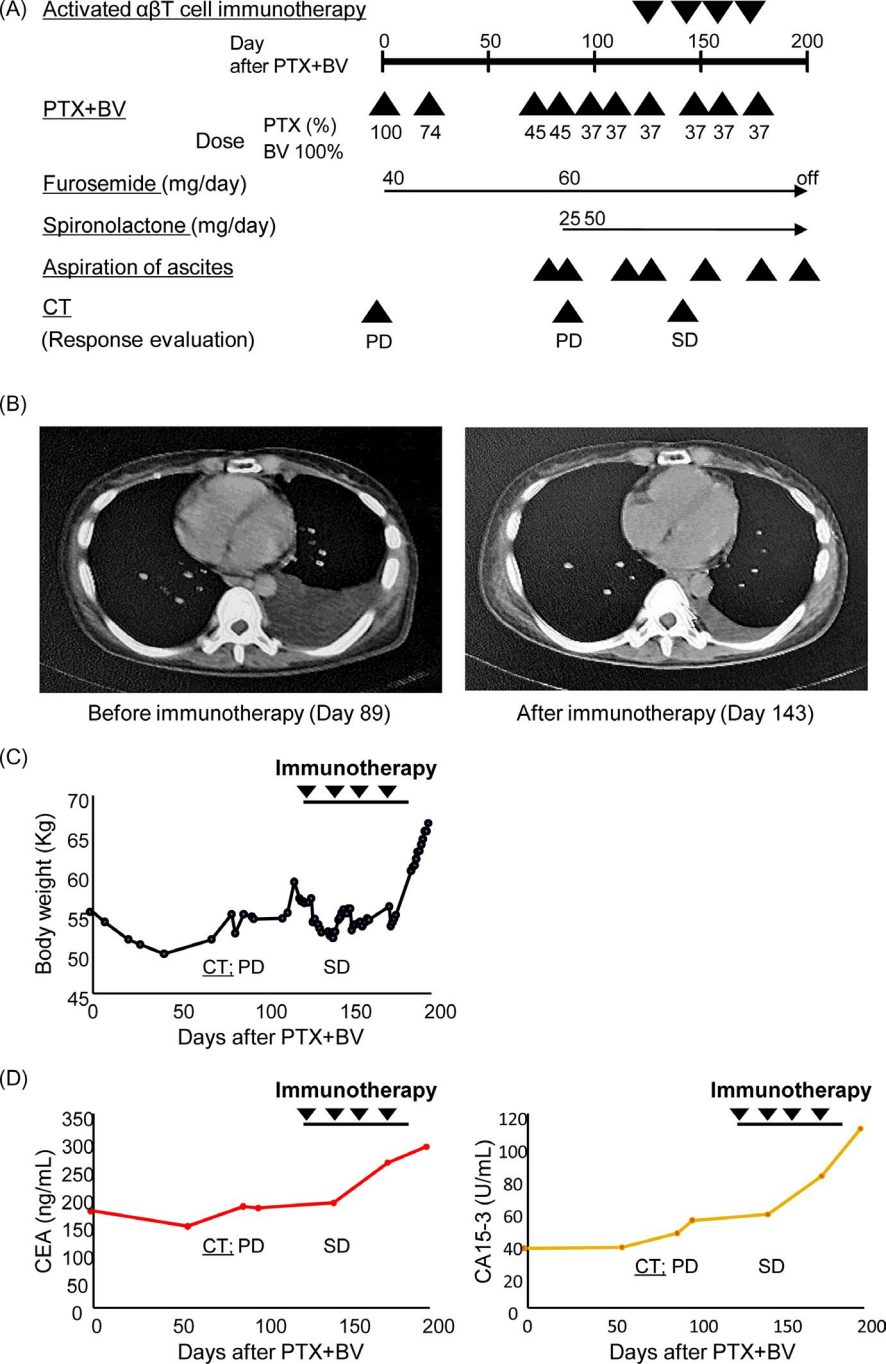
Treatments and clinical course. A, Therapeutic course. B, CT before ATI (left) and after second ATI (right). C, Body weight. D, Tumor markers (left, CEA; right, CA15‐3). Abbreviations: BV, bevacizumab; CT, computed tomography; PD, progressive disease; PTX, paclitaxel; SD, stable disease

She wanted immunotherapy as her next treatment rather than chemotherapy including eribulin and oral 5‐fluorouracil derivative S‐1. We performed ATI combined with PTX + BV, expecting synergistic effects because BV can restore antitumor immunity by neutralizing VEGF and improving immune cell delivery to tumors through vessel normalization.^4^ ATI combined with biweekly PTX + BV began on Day 125 at the Center for Advanced Medical Innovation, Kyushu University (Fukuoka, Japan), as described elsewhere.^3^ Briefly, peripheral blood mononuclear cells (PBMCs) were cultured with immobilized anti‐CD3 antibody and IL‐2 for 2 weeks, and then 5 × 10^9^ lymphocytes were harvested. Expanded lymphocytes were infused intravenously and injections repeated every 2 weeks. Cultured lymphocytes were mainly activated αβ T cells (CD3+ TCRαβ+; Figure 2A).

**FIGURE 2:**
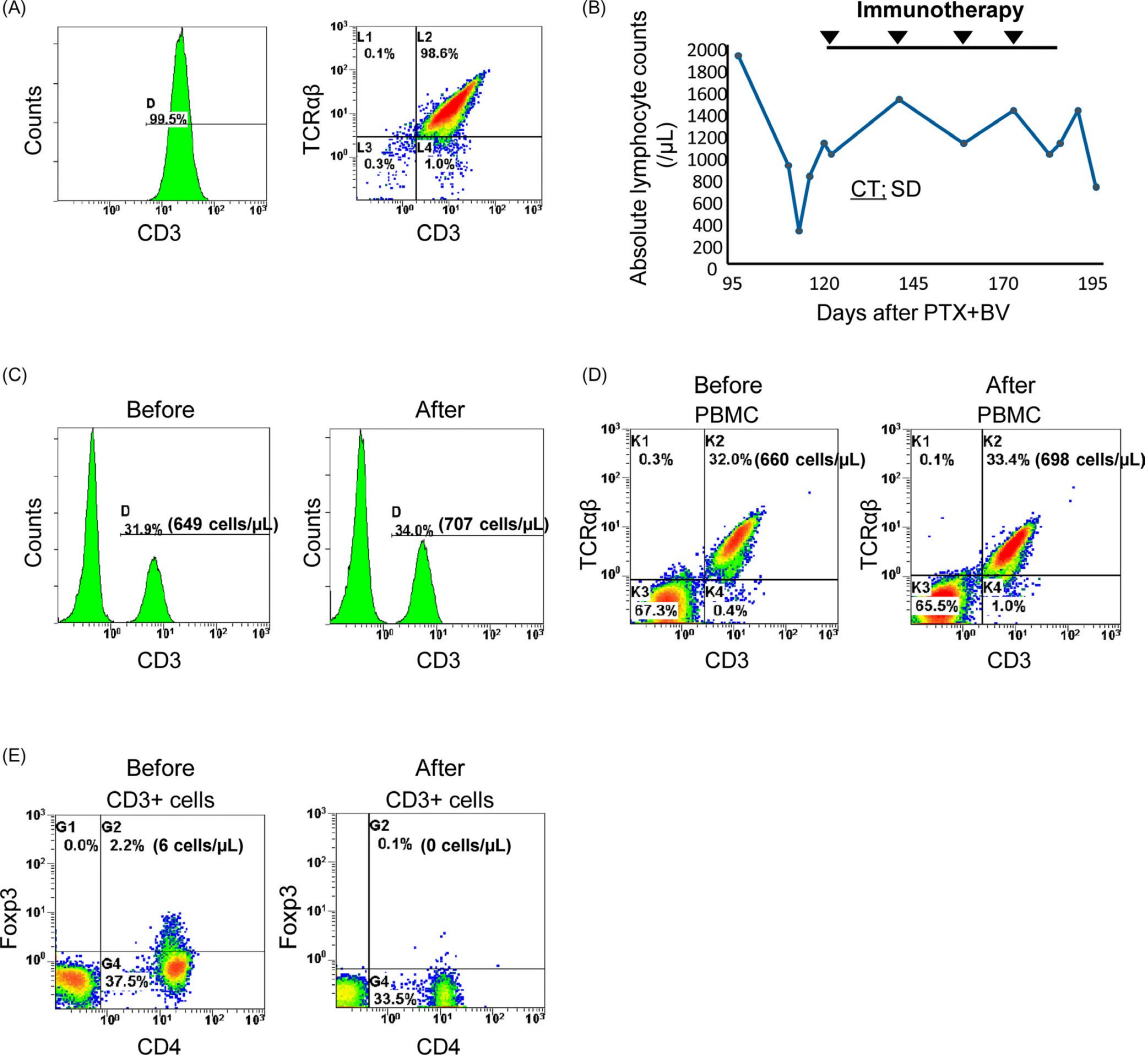
Changes in PBMCs with ATI. A, FACS of activated αβ T cells from the patient; left, histogram of CD3+ cells; right; dot plot of CD3+ cells. B, Changes in absolute lymphocyte counts. C, Histogram of CD3+ PBMCs by FACS analysis before ATI (left) and after 3rd ATI (right). D, FACS dot plots of PBMCs before ATI (left) and after 3rd ATI (right). E, FACS dot plots of CD3+ PBMCs before ATI (left) and after 3rd ATI (right). Abbreviations: CT, computed tomography; FACS, fluorescence‐activated cell sorting; PBMC, peripheral blood mononuclear cell

On Day 143, CT revealed stable disease with a marked decrease in pleuritis and no metastatic tumor progression (Figure 1B). Furthermore, the number of required ascites aspirations decreased without increasing body weight, and breathing improved (Figure 1A,C). Additionally, the rate of increase of tumor markers CEA and CA15‐3 did not change after 1st ATI (Figure 1D). These results suggest that this combination therapy had antitumor activity and improved cancer‐related symptoms without adverse events in metastatic HR‐positive BC resistant to anthracycline/taxane chemotherapy.

Fluorescence‐activated cell sorting of PBMCs performed at MEDINET (Tokyo, Japan) assessed changes in immune cell numbers following ATI. As expected, numbers of T cells (CD3+) and αβ T cells (CD3+/TCRαβ+) increased slightly during ATI from 649/µL (31.9%) and 660/µL (32.0%) to 707/µL (34.0%) and 698/µL (33.4%), respectively (Figure 2B‐D). Numbers of Foxp3+ CD4+ regulatory T cells (Tregs) decreased from 6/µL (2.2%) to 0/µL (0.1%) during ATI (Figure 2E) as previously reported.^5^ Increased effector αβ T cells and decreased immunosuppressor Tregs may explain the clinical effects of this therapy.^5^ Furthermore, BV might enhance ATI by inhibiting VEGF immunosuppressive effects and improving immune cell delivery to tumor by vessel normalization,^4^ as found in hepatocellular carcinoma following anti‐PD‐L1 plus BV.^6^

In February 2020 after her 4th ATI, she was admitted to hospital for general fatigue and unstable vital signs and switched to terminal care.

Here, we report the first case of anthracycline‐ and taxane‐resistant HR‐positive BC with pleural effusion and ascites who safely received ATI combined with PTX + BV with clinical benefit. This combination may be useful for advanced BC patients, although a biomarker to predict clinical response should be required for clinical application.

## CONFLICT OF INTEREST

All authors declare no conflicts of interest.
